# The emerging and diverse roles of F-box proteins in spermatogenesis and male infertility

**DOI:** 10.1186/s13619-024-00196-9

**Published:** 2024-06-26

**Authors:** Xuan Zhuang, Jun Ruan, Canquan Zhou, Zhiming Li

**Affiliations:** 1 Institute of Reproductive Health, Tongji Medical College, Huazhong University of Science and Technology, Wuhan, Hubei 430030 China; 2 Department of Clinical Medicine, Fujian Medical University, Fuzhou, Fujian 363000 China; 3 Department of Urology, the First Affiliated Hospital of Xiamen University, Xiamen, Fujian 361003 China; 4 College of Life Sciences, Central China Normal University, Wuhan, Hubei 430079 China; 5 Guangdong Provincial Key Laboratory of Reproductive Medicine, Guangdong Provincial Clinical Research Center for obstetrical and gynecological diseases, Center for Reproductive Medicine and Department of Gynecology & Obstetrics, the First Affiliated Hospital of Sun Yat-Sen University, Guangzhou, 510080 China

**Keywords:** F-box proteins, Ubiquitylation, Spermatogenesis, Male infertility, Male germ cells

## Abstract

F-box proteins play essential roles in various cellular processes of spermatogenesis by means of ubiquitylation and subsequent target protein degradation. They are the substrate-recognition subunits of SKP1–cullin 1–F-box protein (SCF) E3 ligase complexes. Dysregulation of F‑box protein‑mediated proteolysis could lead to male infertility in humans and mice. The emerging studies revealed the physiological function, pathological evidence, and biochemical substrates of F-box proteins in the development of male germ cells, which urging us to review the current understanding of how F‑box proteins contribute to spermatogenesis. More functional and mechanistic study will be helpful to define the roles of F-box protein in spermatogenesis, which will pave the way for the logical design of F-box protein-targeted diagnosis and therapies for male infertility, as the spermatogenic role of many F-box proteins remains elusive.

## Background

**Fig. 1:**
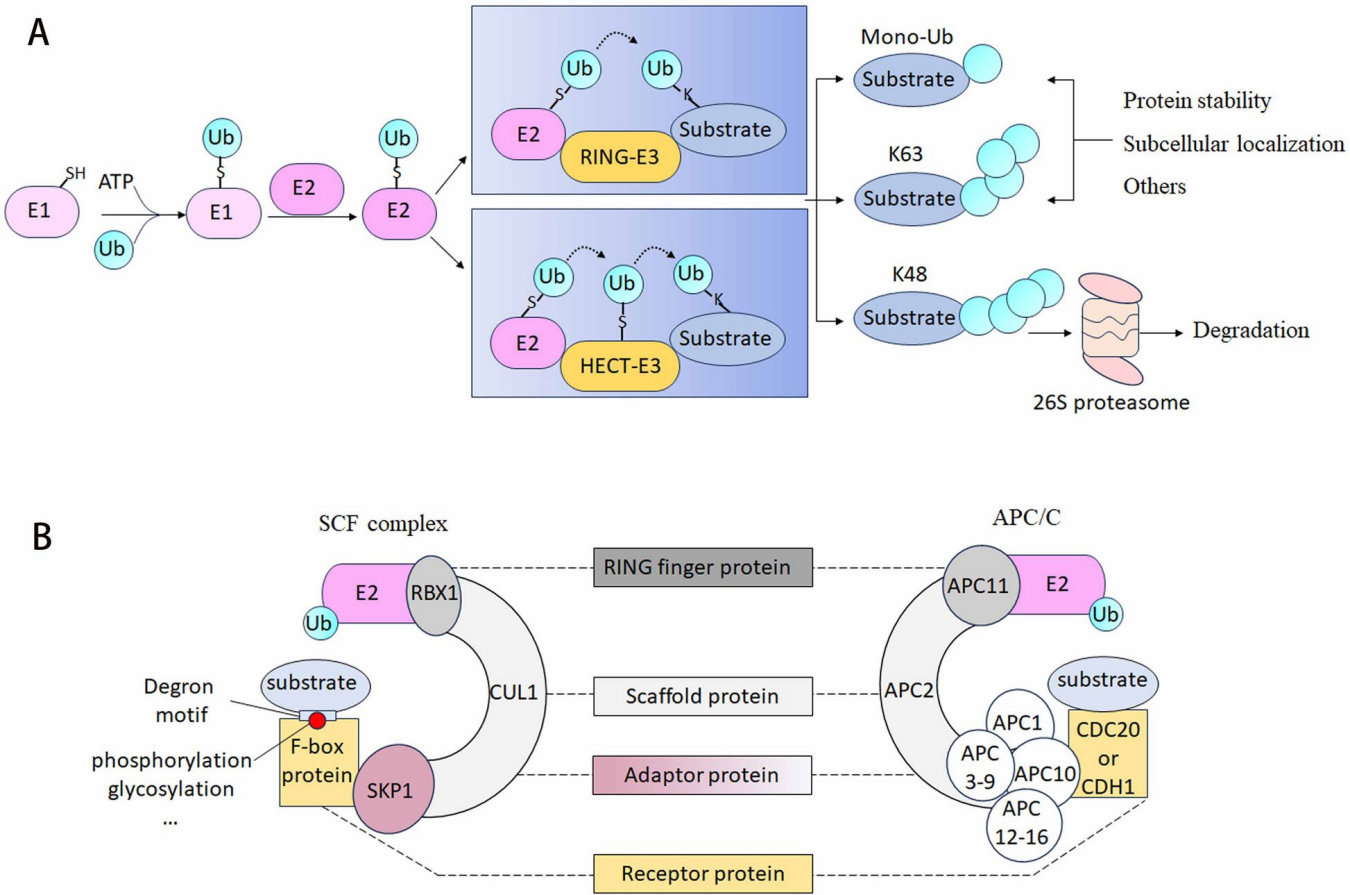
Ubiquitin-mediated degradation. **A** Overview of the cascade process of ubiquitination. Ubiquitination is a cascade process of posttranslational modification catalyzed by three key enzymes. Ubiquitin (Ub) is activated in an ATP-dependent manner by the E1 activating enzyme and then transferred to the E2 conjugating enzyme. Finally, ubiquitin is covalently attached to the substrate via the E3 ligase. Generally, K48-linked poly-ubiquitination is involved in protein stability control, while monoubiquitylation and K48-linked poly-ubiquitination are linked to the regulation of subcellular localization and gene expression. **B** The structural similarity of the SCF and the APC/C. The SCF and APC/C E3 ligases are two members of the Cullin-RING ligase (CRL) superfamily. CUL1 and APC2 are the scaffold proteins. They bind to the RING finger protein RBX1 or APC11, which recruits the E2 ubiquitin conjugating enzyme. On their other end, scaffold proteins connect to the substrate recognition subunit F-box proteins, or CDH1 and CDC20, via the adaptor protein, such as SKP1. Canonically, F-box proteins recognize the substrate by the degron modifications, such as phosphorylation and glycosylation

The ubiquitin–proteasome system (UPS) is responsible for the degradation of proteins by ubiquitylation, a post-translational modification that regulates various physiological functions including transcription (Wang et al. 2022), apoptosis (Sharma and Trivedi 2020), proliferation (Kim et al. 2023), and germ cell development (Xiong et al., 2022). The UPS exerts its biological functions through a cascade of three enzymatic reactions, which are catalyzed by the ubiquitin-activating E1 enzyme, the ubiquitin-conjugating E2 enzyme, and the ubiquitin-protein E3 ligase (Fig. 1). The substrate specificity for ubiquitylation and subsequent degradation is determined by E3 ligases. The largest family among the E3 ubiquitin ligases is cullin–RING E3 ligase (CRL) (Duan and Pagano 2021). CRL1 is also known as the SKP1–cullin1–F-box protein (SCF), which is the most well-characterized family member. The SCF complex includes the variable F-box proteins that provide substrate specificity by specifically targeting a particular substrate for ubiquitylation, and the invariant components SKP1, RBX1, and cullin1 (Horn-Ghetko et al. 2021). In addition to SCF, another multi-component E3 ligase named as anaphase-promoting complex/cyclosome (APC/C) has also been demonstrated to be an essential regulator of protein degradation (Yatskevich et al. 2021). APC/C is structurally comparable to SCF by containing the invariable subunits APC1 (RBX1-related RING-finger protein), APC2 (a cullin-like scaffolding protein), and the variable component CDH1 or CDC20, which determine substrate-specificity and play roles similar to F-box proteins.

**Fig. 2:**
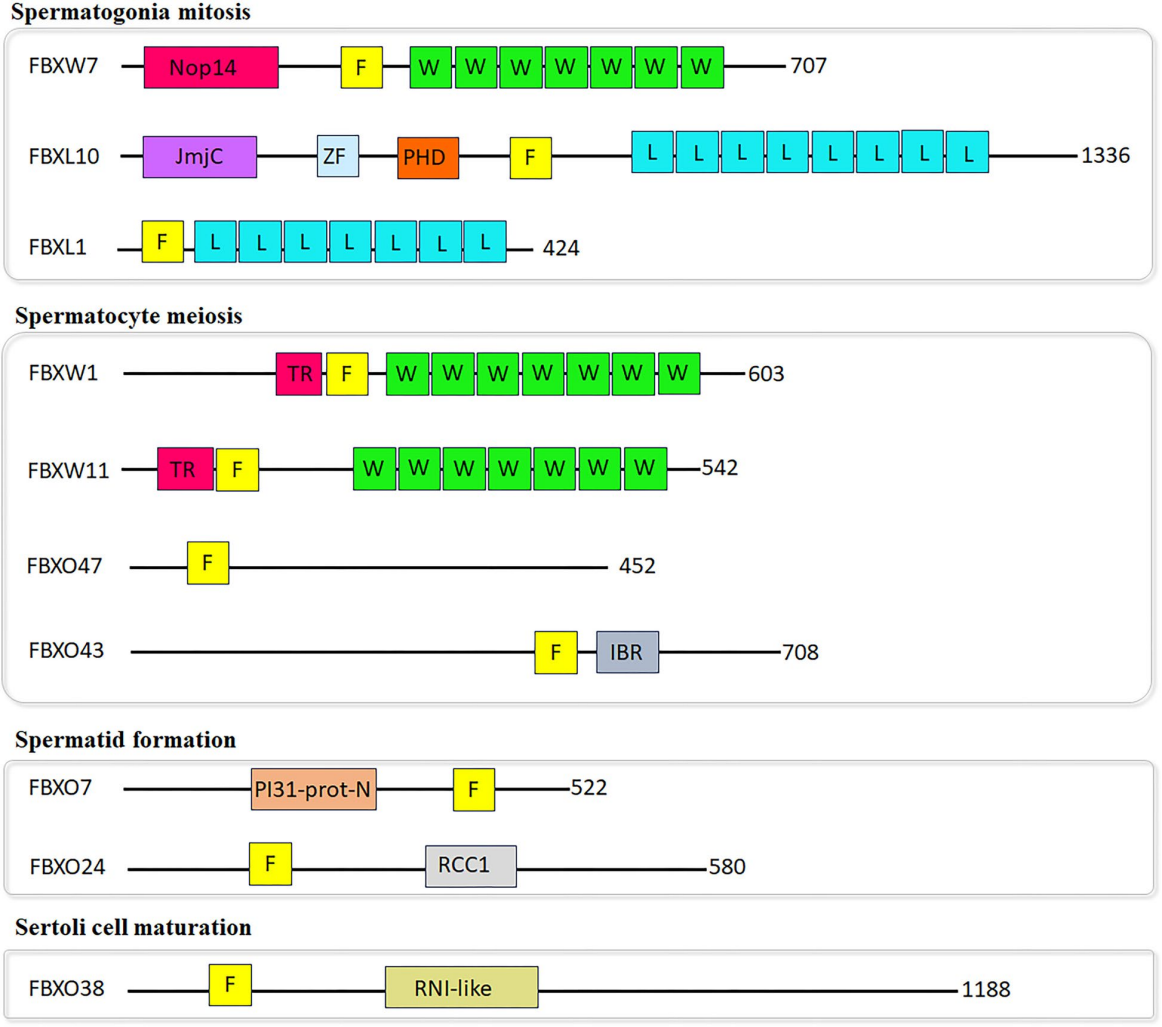
Domain structures of mammalian F-box proteins. The F-box proteins are grouped by the biological functions (spermatogonia mitosis, spermatocyte meiosis, spermatids formation, and Sertoli cell maturation) in the context of spermatogenesis that have been documented by available mouse models. *F* F‑box motif, *W* WD40 repeat motif, *L* leucine‑rich repeat, *Nop14* NOP14‑like family domain, *PI31‑prot‑N* PI31 proteasome regulator amino‑terminal domain, *Tr* D domain of β‑TRCP, *IBR* in between ring fingers domain, *JmjC* Jumonji C domain, *RCC1* regulator of chromosome condensation 1, *RNI-like* RNase inhibitor (RNI)-like, *PHD* plant homeodomain. The amino acid length of human F-box proteins is indicated

Each F-box protein is composed of at least two major functional domains: the F-box motif, a protein–protein interaction domain, which recruits F-box proteins into the SCF complex via direct binding with SKP1, and various carboxy-terminal domains that bind to specific substrates. Based on the distinct substrate recognition domains, F-box proteins are classified into three subclasses: WD40 repeats (FBXW), leucine-rich repeats (FBXL), and other uncharacterized domains (FBXO) (Fig. 2).

How do substrates become recognized by F-box proteins? They often target certain degrons in the substrates. The interaction of the substrates with the corresponding F-box proteins usually depends on the appropriate post-translational modifications of the degrons (Skaar et al. 2013). For instance, the conserved phosphodegron sequence is commonly found in FBXW7 substrates (Nash et al. 2001). Phosphorylation of this motif is necessary for FBXW7 to identify and ubiquitylate its substrates. In addition to phosphorylation, F-box proteins are capable of identifying degrons that are modified by glycosylation or mannose oligosaccharides. For example, FBXO6 recognizes T cell receptor α-chain by the glycosylated degron (Yoshida et al. 2003), and FBXO2 binds precursor β1 integrin containing the N-linked high-mannose oligosaccharides (Yoshida et al. 2002). Unexpectedly, phosphorylation is also present to prevent the substrate degradation by FBXL2 (Kuchay et al. 2013) and FBXO11 (Abbas et al. 2013). Additionally, FBXO1 recognizes and ubiquitylates CP110 (centriolar coiled-coil protein 110) and RRM2 (ribonucleotide reductase regulatory subunit M2) via an unmodified degron (D'Angiolella et al. 2010). Several ways are reported to function in recognition of substrates by F‑box proteins, including canonical phosphodegron, cofactor-dependent degron, restricted degron access, priming phosphorylation, unmodified degrons, inducible and non-covalent degron, domain-based recognition, and inhibited degron (Skaar et al. 2013).

An increasing number of studies reveal that F-box protein is crucial for protein turnover in spermatogenesis. Spermatogenesis is a complex and well-organized process in which a diploid spermatogonium develops into a specialized haploid male gamete, through successive rounds of cell division and differentiation. Generally, spermatogenesis involves three phases of development: (1) mitosis, spermatogonia stem cells undergo self-renew and differentiate; (2) meiosis, primary spermatocytes go through two meiotic cycles, producing haploid spermatids; and (3) spermiogenesis, extensive cell morphological alterations in the spermatid, differentiating into highly specialized spermatozoa (Li et al. 2022). The majority of the testis mass is made up of seminiferous tubules, which are responsible for producing germ cells. Sertoli cells function to nourish the developing germ cells, which line the tubule. The basal layer of seminiferous tubules contains the differentiation progeny of spermatogonia. These germ cells go to the abluminal compartment through meiosis, developing into spermatocytes, after which they translocate to the second layer. Post-meiotic spermatids migrate through the third and fourth stratified cell layers and are finally released into the lumen. The duration of spermatogonia development to the sperm takes 35 days (Clermont 1972). The full process of spermatogenesis in humans takes around 74 days (Griswold 2016).

**Fig. 3:**
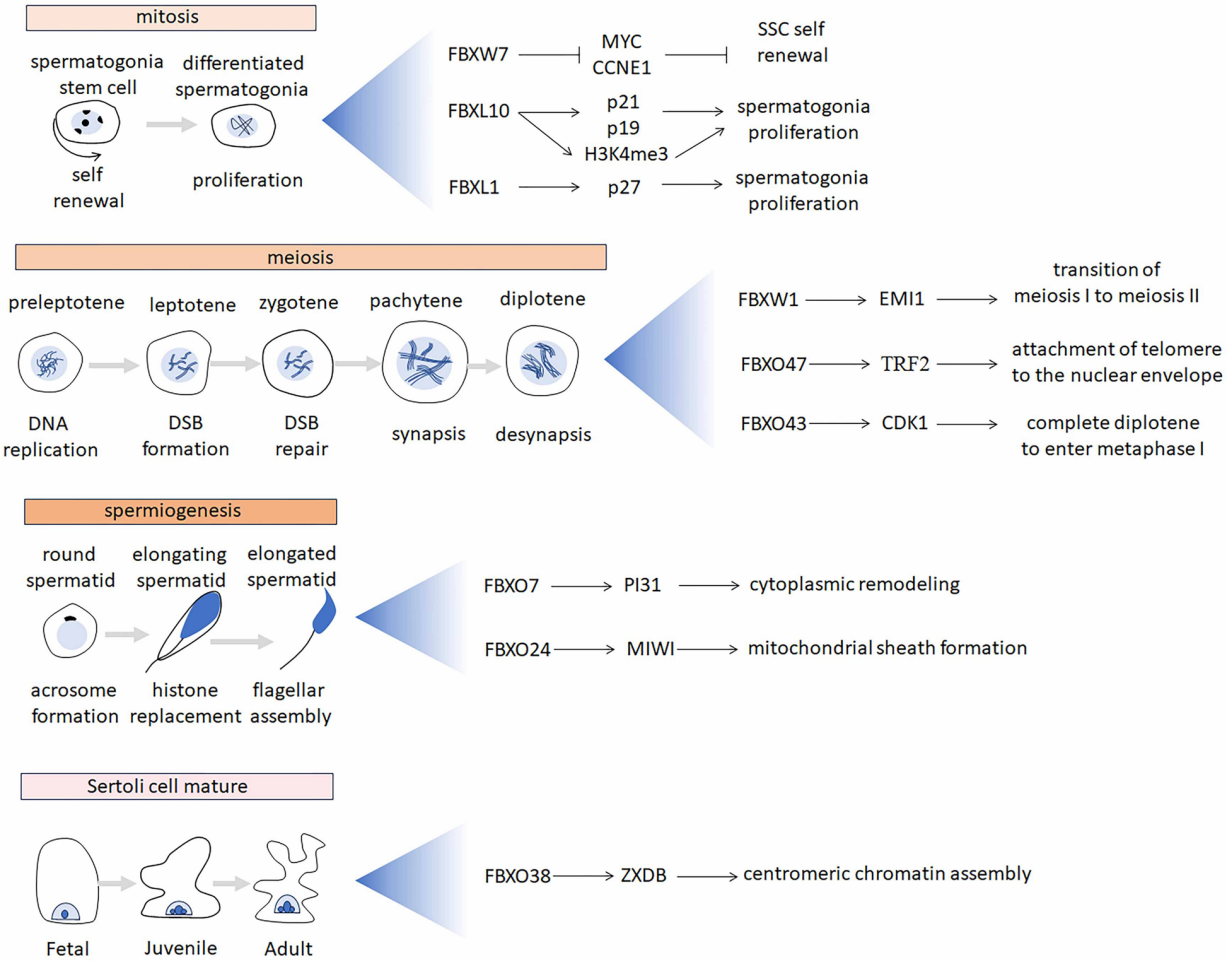
Overview of F-box proteins functions during mammalian spermatogenesis. F-box proteins are expressed in the male germ cell (spermatogonia, spermatocyte, spermatid) and the somatic cell (Sertoli cell). F‑box proteins are divided into four categories according to their roles in the development of male germ cells. Spermatogenesis consists of the mitotic division of spermatogonia, meiosis of spermatocytes, and post-meiotic differentiation of spermatids (spermiogenesis), processes tightly controlled by hormones and growth factors secreted by Sertoli cells. The hallmark events are marked below the cells. SSC, spermatogonia stem cell. DSB, double strand breaking. F-box proteins and their targets are displayed at the right of the biological process

In 1999, Michele Pagano et al. reported the identification of a family of F-box proteins in humans (Cenciarelli et al. 1999). Over the past 25 years, a great deal of effort has been devoted to discovering the substrates that are ubiquitylated by a given F-box protein and thereby deciphering its physiological functions. Notably, there are ongoing studies that aim to generate animal models to understand the physiological function of F-box proteins in the testis, or that aim to explore a connection between the genetic alterations of F-box proteins and human spermatogenetic failure. In this paper, we provide a comprehensive summary of the roles of the F-box proteins in spermatogenesis based on the available data (Table 1). According to the process of spermatogenesis, the physiological roles of F-box proteins are classified into four categories: mitotic division of spermatogonia, meiosis of spermatocytes, post-meiotic differentiation of spermatids, and Sertoli cell maturation (Fig. 3).

**Table 1:** The phenotypes of the loss of the F-box genes in mice or humans

Gene	Knockout phenotypes	Gene repression method	Substrates	Species	Reference
***Fbxw7*** **(*Ago*;* Cdc4*)**	reduced meiotic cells and germ cells; enhanced SSC colonization; accumulation of undifferentiated spermatogonia;	*Stra8*-Cre-LoxP recombination system	MYC CCNE1	mice	Kanatsu-Shinohara et al. 2014
***Fbxl10*** **(*Kdm2b* / *Jhdm1b*)**	slower growth of SSC; a progressive loss of spermatogonia with age;	Gene-targeting vector system	P21 P19	mice	Ozawa et al. 2016
***Fbxl1*** **(*Skp2*)**	reduced male fertility; a progressive loss of spermatogonia with age;	Gene-targeting vector system	P27	mice	Fotovati et al. 2006
***Fbxw1*** **(β-Trcp1,** ***Fbw1a*)**	reduced male fertility; accumulation of methaphase I spermatocytes;	Gene-targeting vector system	EMI1	mice	Guardavaccaro et al. 2003
***Fbxo47***	male infertility; uncompleted synaptonemal complex; lack crossovers;	CRISPR/Cas9	TRF2	mice	Hua et al. 2019; Tanno et al. 2022
***Fbxo43*** **(*Emi2*, *Xerp1*)**	male infertility; arrest at diplotene of prophase I;	*Stra8*-Cre-LoxP recombination system	CDK1	mice	Gopinathan et al. 2017
	male infertility; non-obstructive azoospermia;	homozygous mutation (c.1747CT, p.Q583X)	-	humans	Wu et al. 2022
	male infertility; teratozoospermia;	homozygous mutation (C1991T, p.G664D)	-	humans	Ma et al. 2019
***Fbxo7*** **(*Park15*, *Pkps*)**	male infertility; reduced sperm; phagocytosed sperm;	LacZ insertion	PI31	mice	Rathje et al. 2019
***Fbxo24*** **(*Fbx24*)**	male infertility; defective sperm mitochondrial sheath; aberrant sperm histone retention; incomplete sperm axonemes;	CRISPR/Cas9	MIWI	mice	Kaneda et al. 2023
***Fbxo38*** **(*Sp329*,** ***Moka*)**	reduced fertility; smaller testis; immature Sertoli cell;	CRISPR/Cas9	ZXDB	mice	Dibus et al. 2022

## F-box proteins function in spermatogonia mitosis

### FBXW7

F-box and WD-40 domain protein 7 (FBXW7, also called AGO, CDC4), is essential for the control of the proliferation of stem cells and progenitors (Takeishi and Nakayama 2014). FBXW7 is specifically expressed in the undifferentiated spermatogonia compartment. *Fbxw7* deletion in the testis is achieved by crossing the floxed *Fbxw7* allele (*Fbxw7*^f/f^) homozygous mice with retinoic acid gene 8 (*Stra8*)-Cre transgenic mice which express Cre recombinase specifically in spermatogonia from 3 days postpartum (Kanatsu-Shinohara et al. 2014). Histological examination of the 2-month-old *Fbxw7*^f/f^*Stra8*-Cre testes showed that the germ cells were significantly reduced in the mutant testes. *Fbxw7* deletion leads to the increased proliferation of undifferentiated spermatogonia (Kanatsu-Shinohara et al. 2014), suggesting it may be a negative regulator of spermatogonial stem cell (SSC) self-renewal. FBXW7 is a well-established tumor suppressor, and its expression needs to be tightly controlled in nontransformed cells. It is consistent with the result that FBXW7 is restricted to undifferentiated spermatogonia in the testis. *Fbxw7* deficiency caused the increased expression of the myelocytomatosis oncogene (MYC) and cyclin E1 (CCNE1), which are regulators of transcription and cell cycle, respectively. Notably, inhibitors that target FBXW7 have shown promising potential in anticancer therapies (Naseem et al. 2023). Modulation of FBXW7 function by small molecules may be useful for enhancement of SSC self-renewal.

### FBXL10

F-box and leucine-rich repeat protein 10 (FBXL10, also called KDM2B and JHDM1B), also a histone lysine demethylase possessing the histone lysine demethylase catalytic JmjC domain, and catalyzes the demethylation of H3K4 (Janzer et al. 2012) and H3K36 (He et al. 2008). *Fbxl10* has two different isoforms, a long isoform contains the catalytic JmjC domain for histone demethylation, and a short-form isoform that lacks the JmjC domain although other domains are the same as in the long isoform. *Fbxl10* long isoform knockout (*Fbxl10*^ΔJ/ΔJ^) caused a significant increase in the occurrence of neural tube defects during fetal development, leading to perinatal mortality (Fukuda et al. 2011). Although some *Fbxl10*^ΔJ/ΔJ^ mice can live to maturity, the epididymis of mutant males has significantly fewer sperm (Ozawa et al. 2016). The proliferation rate of cultured *Fbxl10*^ΔJ/ΔJ^ spermatogonia was significantly slower than wild-type (WT) cells. *Fbxl10*^ΔJ/ΔJ^ caused a progressive degeneration of spermatogenesis in the seminiferous tubules. The cellular senescence markers p21 and p19 mRNA were increased in cultured *Fbxl10*^ΔJ/ΔJ^ spermatogonia. p21 and p19 are important regulators of cell cycle checkpoints, which can prevent cell cycle progression from the G1 to S phase (Capparelli et al. 2012). These data suggest that FBXL10 ensures long-term sustainable spermatogenesis via regulating the cell cycle. Notably, *Fbxl10*^ΔJ/ΔJ^ caused a drastic alteration in the distribution of H3K4me3 in testicular germ cells. H3K4 is closely associated with transcriptional activation and the proper regulation of H3K4 is important for fertility (Lambrot et al. 2021). For example, gene knockout of the H3K4 methyltransferase causes male infertility in mice, accompanied by a reduced level of H3K4me3 in testicular germ cells (Hayashi et al. 2005). The long isoform of *Fbxl10* sustains spermatogenesis for a long period through both E3 ligase activity and histone modification.

### FBXL1

F-box and leucine-rich repeat protein 1 (FBXL1, also named SKP2), is an important regulator of the S phase of the cell cycle. *Fbxl1* deficient mice are viable, but their hepatocyte cells have noticeably larger nuclei with polyploidy and many centrosomes (Nakayama et al. 2000). *Fbxl1* deficient male mice had lower fertility (Fotovati et al. 2006). A progressive loss of spermatogonia with age was observed in *Fbxl1* deficient testis, with only Sertoli cells remaining in the seminiferous tubules. Additionally, a substantial number of polyploid cells was observed in the semen of *Fbxl1* deficient males. Polyploidy and consequent apoptosis are likely to be responsible for the formation of giant multinucleated structures in the seminiferous epithelium, and the detachment of spermatogonia and spermatocytes from the tubules of *Fbxl1* deficient males. The expression of p27 in the embryonic testis at the 15.5 days postcoitum was significantly increased in *Fbxl1* deficient mice. p27 is a cyclin-dependent kinase (CDK) inhibitor, which negatively regulates the progression of cell cycle. FBXL1 was demonstrated as a key cell cycle regulator as it mediates ubiquitylation and subsequent degradation p27 (Carrano et al. 1999). It has been showed the lack of p27 degradation results in suppression of CDK1 and consequent a failure of entry into the M phase (Nakayama et al. 2004). *Fbxl1* absence in the testis leads to germ cell aneuploidy may be through a similar mechanism.

## F-box proteins function in spermatocyte meiosis

### FBXW1

F-box/WD repeat-containing protein 1 (FBXW1, also known as FBXW1A or βTrCP1), controls meiotic and mitotic events. In mice, loss of *Fbxw1* results in impaired spermatogenesis and decreased fertility (Guardavaccaro et al. 2003). In *Fbxw1* deficient testis, some spermatocytes divide improperly and finally produce multinucleated spermatids, while another proportion advances slowly through meiosis showing the accumulating metaphase I spermatocytes in the testis. There are significantly lower of spermatids and spermatozoa in the testis of *Fbxw1* deficient mice. An accumulation of EMI1 (early mitotic inhibitor 1) was only observed in the testis of *Fbxw1* deficient, but not in other organs. The defect of ubiquitination-mediated EMI1 degradation was demonstrated in mouse embryonic fibroblast (MEF) of *Fbxw1* deficient mice, indicating that EMI1 is a direct substrate of FBXW1. The upstream signaling pathway of FBXW1 has been defined in HEK293T cells. FBXW1 is the target of extracellular signal-regulated kinase 2 (ERK2), which interacted and phosphorylated with two independent ERK docking sites located in the F-box domain and linker domain on FBXW1 (Lee et al. 2021). In addition, FBXW1 and FBXW11 (also known as βTrCP2) are two paralogs of βTrCP in the mammalian cells. *Fbxw11* deficient mice died during embryogenesis as a result of abnormal development of extraembryonic tissue (Nakagawa et al. 2015). Targeted deletion of *Fbxw11* in male germ cells by *Stra8*-Cre on a background of whole-body *Fbxw1* knockout resulted in sterility due to a lack of mature sperm (Nakagawa et al. 2017). The meiotic cells were essentially absent and the tubule lumen was largely empty in the double-knockout (DKO) testis. FBXW1 and FBXW11 are considered to be functionally redundant and indistinguishable. It is consistent with the findings that β-TrCP1/2 DKO male mice are sterile, while β-TrCP1 KO males and β-TrCP2 CKO males are not.

### FBXO47

F-box only protein 47 (FBXO47), regulates telomere-inner nuclear envelope integration. In *Caenorhabditis elegans*, mutation of prominin 1 (*prom-1*), the homolog of *Fbxo47*, caused impaired homologous synapsis during meiotic prophase I (Jantsch et al. 2007). In mice, FBXO47 is predominantly expressed in the meiotic spermatocyte and locates on the nuclear surface at the onset of meiosis. The testes weight of *Fbxo47* knockout males is reduced, only with abnormal spermatocyte-like cells in some seminiferous tubules (Hua et al. 2019). The spermatocytes of *Fbxo47* knockout mice are arrested at a late-zygotene stage, marked by incomplete chromosomal synapsis while having the chromosome pairing and the production of synaptonemal complex axial elements. Meiotic recombination involves the repair of DNA double-strand breaks. The late-zygotene arrest *Fbxo47* knockout spermatocytes still remained γH2AX-positive at the terminal of autosome, indicating an impairment of DNA double-strand repair. Recently, Nobuhiro Tanno et al. found that *Fbxo47* knockout pachytene and diplotene-like spermatocytes did not exhibit XY bodies, indicating that FBXO47 is also critical for the formation of XY body (Tanno et al. 2022). During the onset of meiosis, chromosomes move along the nuclear envelope and telomeres attach to the nuclear envelope. However, *Fbxo47* knockout spermatocytes are unable to attached to the nuclear envelope due to the disruption of the interaction with telomere restriction fragment 2 (TRF2) and telomere restriction fragment 1 (TRF1). The expression of telomeres protein TRF2 and TRF1 are decreased in the *Fbxo47* knockout testis. These results indicate that FBXO47 is necessary for telomere function during the bouquet stage when the meiotic telomeres attach to the inner nuclear membrane.

### FBXO43

F-box only protein 43 (FBXO43, also known as EMI2 or XERP1), inhibits APC/C activity. *Fbxo43* knockout mice displayed a normal lifespan and infertility (Gopinathan et al. 2017). Histopathological analysis revealed a complete absence of spermatids in *Fbxo43* knockout testes. *Fbxo43* deficient spermatocytes arrest at early diplotene of prophase I. The chromosomes of *Fbxo43* deficient mice displayed normal SYCP1 (synaptonemal complex protein 1) staining at pachytene and no SYCP1 was detected in diplotene. The disassembly of the synaptonemal complex following pachytene is observed in *Fbxo43* deficient spermatocytes. These results indicate that *Fbxo43* deficient spermatocytes progress normally through the steps preceding diplotene but are unable to complete diplotene to enter metaphase I. Moreover, *Fbxo43* knockout testes exhibited decreased kinase activity of CDK1 and cyclin B1, but the protein and mRNA expression did not change. FBXO43 is expressed in spermatocytes with the highest level but also exists in spermatids and sperm. It implies that FBXO43 plays important roles in the meiosis and the post-meiotic process. In the patients with non-obstructive azoospermia (NOA) from a Chinese consanguineous family, a homozygous nonsense mutation (c.1747C > T, p.Q583X) of FBXO43 was found to be the cause of meiotic spermatocyte arrest at early diplotene of prophase I (Wu et al. 2022). A homozygous nonsynonymous mutation (C1991T, p.G664D) of FBXO43 was identified to be associated with male infertility and teratozoospermia based on the sequence data from two Chinese brothers with consanguineous parents (Ma et al. 2019). This mutation results in abnormal spermatozoa, showing a larger amorphous head and lacunar chromatin defect. Structurally, C1991T, p.G664D is located within the IBR (in between ring fingers) domain of FBXO43, which is involved in changing the protein secondary structure. However, c.1747C > T, p.Q583X is located in the region between F-box and IBR. It is predicted to be a reason for two phenotypes of male infertility.

## F-box proteins function in spermatid formation

### FBXO7

F-box only protein 7 (FBXO7, also known as PARK15 or PKPS), functions in regulating mitophagy and proteasome activity. FBXO7 down-expression mice are generated by a *LacZ* insertion of *Fbxo7* (Randle et al. 2015). The homozygous *Fbxo7*^LacZ/LacZ^ males are sterile, with very few residual sperm in the epididymis of *Fbxo7*^LacZ/LacZ^ mice. The residual sperm were all grossly misshapen, showing abnormal compression of the sperm head. Massive loss of maturing sperm, mis-localization of late-stage spermatids, and phagocytosis of condensing spermatids are observed in the *Fbxo7*^LacZ/LacZ^ testis. The protein level of PI31(Proteasome Inhibitor of 31,000 Daltons), an inhibitor of the 20S proteasome, was significantly reduced in the *Fbxo7*^LacZ/LacZ^ testes. In *Drosophila*, the mutation of nutcracker (*ntc*), the ortholog of *Fbxo7*, results in sterility (Arama et al. 2007). The spermatids of *ntc* mutant flies undergo apoptosis in late spermiogenesis when individualization would normally occur. PI31 activation of the 26S proteasome is essential for sperm differentiation. The expression of PI31 is significantly decreased in *ntc* mutant testes. These results indicate that PI31 requires a stabilizing interaction with FBXO7 to achieve sufficiently high expression levels.

### FBXO24

F-box only protein 24 (FBXO24, also known as PARK15 or PARK15), a testis-enriched F-box protein. Recently we found that many aberrant splicing events were significantly changed in round spermatids of *Fbxo24* deficient mice (Li et al. 2024), producing a great number of differentially expressed genes. The sperm of *Fbxo24* deficient mice showed aberrant histone retention, defective axonemes, and improper mitochondrial coiling along sperm flagella, leading to male sterility. *Fbxo24* deficiency altered the structures of mitochondria and chromatoid bodies (CB) in the round spermatids. Furthermore, we reveal that FBXO24 mediates the degradation of MIWI via K48-linked polyubiquitination, and interacts with the subunits of SCF. Consistent with the increased expression of MIWI in *Fbxo24* knockout testis, *Fbxo24* depletion caused aberrant upregulation of piRNAs, which controls RNA silencing via the formation of an RNA-induced silencing complex. Yuki Kaneda et al. also reported the phenotype of male sterility and impaired sperm motility of *Fbxo24* knockout mice by deleting a distinct gene region (Kaneda et al. 2023). Interestingly, the accumulation of aberrant granules were observed in the sperm of *Fbxo24* knockout mice. They speculated that FBXO24 has a potential role in preventing the accumulation of ribonucleoprotein (RNP) granules in sperm flagella. These data imply that FBXO24 is essential for the formation of the sperm mitochondrial sheath. While infertile men display anomalies in sperm mitochondrial sheaths, the regulators involved in the formation of the sperm mitochondrial sheath are far from our understanding due to a lack of good animal models with the typical phenotype. *Fbxo24* knockout mice remarkably exhibit the defects of sperm midpiece, which is a good model to study the formation of the sperm mitochondrial sheath during spermiogenesis.

## F-box proteins function in Sertoli cell maturation

### FBXO38

F-box only protein 38 (FBXO38, also known as SP329 or MOKA), controls the composition of centromeric chromatin. *Fbxo38* knockout mice have defective spermatogenesis and display growth retarded (Dibus et al. 2022). A significant decrease in the weight of the liver, brain, kidneys, and testes is observed in *Fbxo38* knockout males. The smaller testes of *Fbxo38* knockout males are associated with lower spermatids and sperm production. The testis histological examination revealed that *Fbxo38* knockout males have a delay in the first wave of spermatogenesis from the meiotic entry to sperm production. *Fbxo38* deficient epididymis seems normal in the morphology, but fewer mature sperms were found in the cauda. ZXDB (zinc finger x-linked duplicated B) was stabilized in *Fbxo38* knockout testes, and WT1 (Wilms’ tumor 1, Sertoli cell marker) was decreased. Interestingly, *Fbxo38* mRNA is preferentially expressed in spermatogonia, spermatocytes, and Sertoli cells, while *Zxdb* mRNA is only expressed in Sertoli cells. ZXDB positively controls the level of centromere protein CENP-B (centromere protein B) (Dibus et al. 2022), which may be related to centromere arrangement in the Sertoli cell. *Fbxo38* deficient mice show an apparent shift toward an immature profile in Sertoli cells, suggesting the lack of *Fbxo38* resulted in failed Sertoli cell maturation.

## F-box proteins expression profiles

**Fig. 4:**
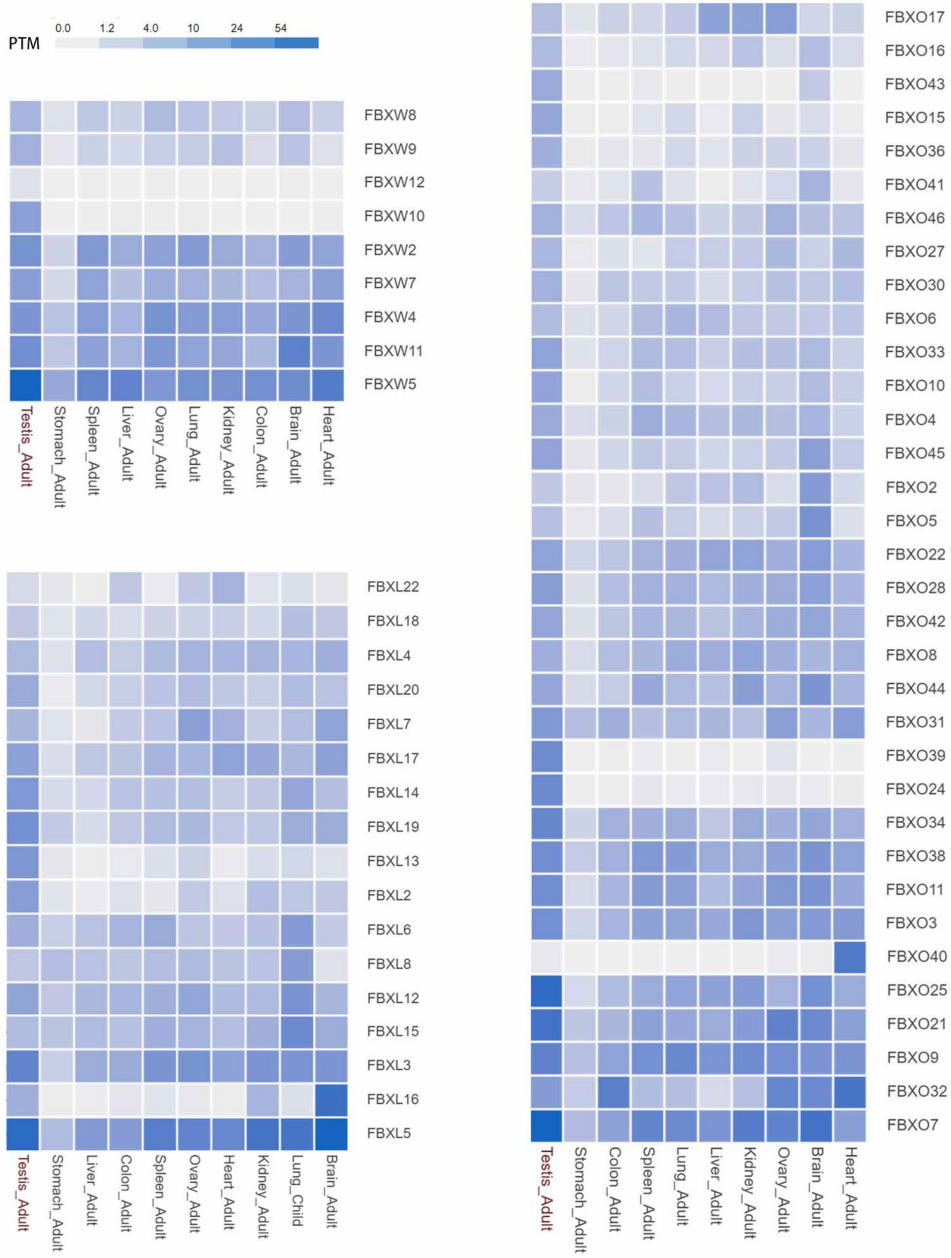
Expression profiles of F-box protein in human normal tissue. The heatmap displays the mRNA intensity of F-box proteins from the FBXW, FBXL, and FBXO subclass. The tissue expression profile is analyzed by TEDD database (Zhou et al. 2023). The color scale represents PTM normalized log2-transformed counts, in which dark blue indicates high expression and light blue indicates low expression. PTM, transcripts per kilobase million

In human normal tissues, the transcripts of each member of the FBXW, FBXL, and FBXO subclass are broadly expressed (Fig. 4). However, the tissue distribution shows that some F-box protein appear to be more selective than others. FBXO40 is expressed at a very low level or not expressed in the testis. Conversely, FBXW12, FBXW10, FBXL13, FBXL2, FBXO16, FBXO43, FBXO15, FBXO36, FBXO39, FBXO24 are found at the highest levels in the testis, suggesting they may be required to spermatogenesis. The physiological functions of many F-box proteins are insufficiently uncharacterized so far. Notably, F-box proteins in the FBXO subclass contain various domains that are not fully clear. Some studies have begun to decipher FBXO biological roles that are attributed to their uncharacterized functional domains (Guo et al. 2014). For the study of a novel F-box protein in the testis, its function can be analyzed by the expression trend in developmental stage testis using the published RNA-seq data of humans and mice.

## F-box proteins of epigenetic regulation

Emerging studies have started to reveal the putative roles of F-box proteins in epigenetic regulation (Table 2). For example, FBXL10 (KDM2A) is a JmjC domain-containing histone demethylase, which catalyzes the demethylation of H3K4 and H3K36 (Vacik et al. 2018). *Fbxl10* deficiency caused drastic alterations of histone methylation in testicular germ cells (Ozawa et al. 2016). In embryonic stem cells (ESC), FBXL10 recruits polycomb repressive complex 1 (PRC1) to CpG islands and regulates H2A ubiquitylation (Wu et al. 2013). Similarly, FBXL11 also has a JmjC domain and plays an essential role in the regulation of histone ubiquitination and DNA methylation (Dhar et al. 2014; Kawakami et al. 2015; Wagner et al. 2013). In addition to directly binding chromatin to mediate epigenetic modifications, F-box proteins also indirectly influence epigenetics through targeting of the regulators. FBXW1 maintains DNA methylation patterns during DNA replication by the proteolysis of UHRF1 (ubiquitin-like with PHD and ring finger domains 1) (Chen et al. 2013). FBXO22 (Tan et al. 2011) and FBXL4 (Van Rechem et al. 2011) regulate the proteolysis of KDM4A, which is a demethylase that targets histone H3K9me2/3 and H3K36me2/3 related to transcriptional activation. FBXO17 ubiquitylates PRMT1 (protein arginine methyltransferase 1) (Lai et al. 2017), a major type I arginine methyltransferase in mammals, methylates both histone and non-histone proteins to regulate transcription, DNA damage response, and signal transduction. Since the function of F-box proteins has not been fully characterized, research in epigenetics is very limited. F-box proteins with epigenetic roles in spermatogenesis need further study.

**Table 2:** F-box proteins in regulation of epigenetics

Protein	Target	Functions	Reference
FBXL10	histone and CpG islands	histone H3 demethylation histone H2A ubiquitylation DNA methylation	He et al. 2008; Janzer et al. 2012; Wu et al. 2013
FBXL11	histone and CpG islands	histone H3 demethylation histone H2A ubiquitylation DNA methylation	Dhar et al. 2014; Kawakami et al. 2015; Wagner et al. 2013
FBXW1	UHRF1	DNA methylation	Chen et al. 2013
FBXO22	KDM4A	KDM4A stability	Tan et al. 2011
FBXL4	KDM4A	KDM4A stability	Van Rechem et al. 2011
FBXL17	PRMT1	PRMT1 stability	Lai et al. 2017

## Conclusions and perspectives

In summary, of around seventy F-box proteins identified in mammals (Mason and Laman 2020), only serval F-box proteins have received male reproductive studies through animal models, including FBXW7, FBXL10, FBXL1, FBXW1, FBXW11, FBXO47, FBXO43, FBXO7, FBXO24, and FBXO38. F-box proteins play critical roles in spermatogenesis mainly via controlling substrate turnover in an E3 ligase activity-dependent way. Remarkably, some studies reported that certain F-box proteins exert their epigenetic functions through non-proteolytic regulation (Liao et al. 2022) (Shen and Spruck 2017). Functional characterization of the F-box protein in the testis will help us understand the precise regulation of protein content in spermatogenesis. F-box proteins need to have more attention in the field of spermatogenesis and male infertility. Although the role of F-box proteins is considered well-characterized in somatic cells, many questions remain in the male germ cells. What is the upstream signaling pathway that controls the activation or inactivation of each given SCF type of E3 ligase? Is there a crosstalk between individual F-box proteins? What are the physiological substrates for many orphan F-box proteins? How can we develop more creative and methodical techniques to identify new substrates for every F-box protein? How can the physiological role of every F-box protein be confirmed? How do the epigenetic regulatory functions of F-box proteins coordinate with their ubiquitylation functions? In order to determine the molecular functions of F-box proteins in the development of testis, we may need to have the aid of reverse genetic methods, such as the whole-body or tissue-specific knockout animal models. Moreover, we also need to develop new methods for the substrate’s identification and mechanisms definition. Future functional studies could be focused on finding the substrates of F-box proteins determining the progression of spermatogenesis, which would be useful in the explanation and treatment of male infertility.

Owing to their interactions with the key players in the testis, F-box proteins are regarded as potential therapeutic targets for male infertility. Recently, chemists have developed a proteolysis-targeting chimeras (PROTACs) technology that can induce targeted protein degradation by the ubiquitin–proteasome system. Before taking the F-box proteins as therapeutic targets, we must make sure the intervention will not cause unexpected consequences by influencing the multifunctional substrates. The drugs are better to target on specific interactions between F-box proteins and substrates in certain types of cells. Identification of upstream regulators and specific compounds of F-box proteins will promote the development of a new therapeutic strategy for future.

## Data Availability

Not applicable.

## Abbreviations

UPS: Ubiquitin–proteasome system
CRL: Cullin–RING E3 ligase
SCF: SKP1–cullin1–F-box protein
APC/C: Anaphase-promoting complex/cyclosome
FBXW: F-box and WD repeat-containing proteins
FBXL: F-box and leucine rich repeat proteins
FBXO: F-box protein
CP110: Centriolar coiled-coil protein 110
RRM2: Ribonucleotide reductase regulatory subunit M2
SSC: Spermatogonial stem cell
MYC: Myelocytomatosis
CCNE1: Cyclin E1
CDK: Cyclin-dependent kinase
EMI1: Early mitotic inhibitor 1
MEF: Mouse embryonic fibroblast
ERK2: Extracellular signal-regulated kinase 2
DKO: Double-knockout
TRF: Telomere restriction fragment
SYCP1: Synaptonemal complex protein 1
NOA: Non-obstructive azoospermia
CB: Chromatoid bodies
RNP: Ribonucleoprotein
ZXDB: Zinc finger x-linked duplicated B
WT1: Wilms’ tumor 1
CENP-B: Centromere protein B
UHRF1: Ubiquitin-like with PHD and ring finger domains 1
PRMT1: Protein arginine methyltransferase 1
PROTAC: Proteolysis-targeting chimeras
